# Differential glycosylation in the envelope glycoprotein enhances DC-SIGN-mediated cell entry of the West African Ebolavirus Makona

**DOI:** 10.1080/22221751.2026.2732697

**Published:** 2026-09-22

**Authors:** Juan García-Bernalt Diego, Fátima Lasala, Joanna Luczkowiak, Clara Delaunay, María A. Noriega, Oscar Malvar, Michel Thépaut, Gonzalo Rivas, Franck Fieschi, Ángeles Canales, José M. Casasnovas, Rafael Delgado

**Affiliations:** a Instituto de Investigación Hospital Universitario 12 de Octubre (Imas12), Madrid, Spain; b Institut de Biologie Structurale, Université Grenoble Alpes, CNRS, CEA, Grenoble, France; c Centro Nacional de Biotecnología, Consejo Superior de Investigaciones Científicas (CSIC), Madrid, Spain; d Instituto de Micro y Nanotecnología, IMN-CNM (CEI UAM+CSIC), Madrid, Spain; e Institut Universitaire de France (IUF), Paris, France; f Dep. Química Orgánica, Facultad de Ciencias Químicas, Universidad Complutense, Madrid, Spain; g Facultad de Medicina, Universidad Complutense, Madrid, Spain

**Keywords:** Ebola, virus transmission, cell entry, glycosylation, DC-SIGN

## Abstract

During the 2013–2016 West African *Ebola virus* (EBOV) Makona epidemic, genomic surveillance identified an A82V substitution in the viral glycoprotein (GP) early in the outbreak. This mutation was associated with increased infectivity and faster viral spread, yet the host factors underlying this enhanced transmission remained unclear. Using a pseudotyped lentiviral system, we show that GP-A82V increases viral binding and infectivity in dendritic cells (DCs) and macrophages, key early and sustained target cells. This enhancement depends on expression of DC-SIGN, a lectin receptor on DCs. Filovirus-like particles carrying GP-V82 displayed accelerated infection kinetics in DC-SIGN–expressing cells. We identified two N-linked glycosylation sites, Ngly257 and Ngly563, that enhanced GP-V82 infectivity through simultaneous engagement of a DC-SIGN dimer. The A82V substitution increased soluble GP binding to DC-SIGN. Nuclear magnetic resonance analysis revealed enrichment of Lewis X (CD15), a known DC-SIGN ligand, in GP-V82. Blocking Lewis X with an anti-CD15 antibody reduced pseudovirus infection, confirming its functional role. These findings identify DC-SIGN as a key host factor contributing to EBOV Makona transmission and highlight viral envelope glycosylation as a mechanism facilitating viral adaptation and enhanced human-to-human spread.

## GRAPHICAL ABSTRACT

**Figure:**
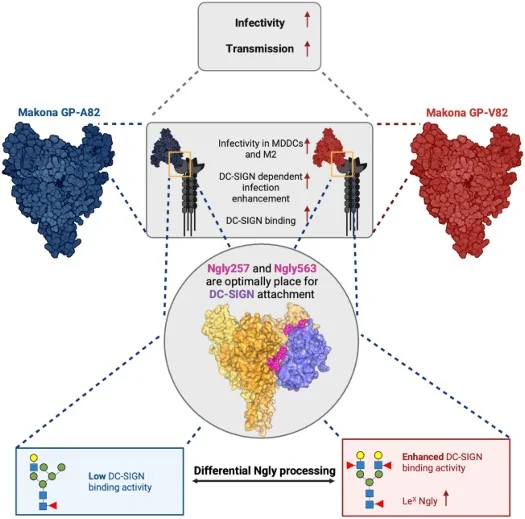

## Introduction

Since its discovery in 1976 in the Democratic Republic of Congo (DRC, formerly known as Zaire) [1], more than twenty EBOV outbreaks have been documented across Africa [2–4]. These outbreaks mainly occurred in remote, poorly communicated areas of Central Africa and typically affected a limited number of individuals (ranging from tens to a few hundred). As a result, they were generally contained by basic public health measures, including patient isolation, contact tracing, and safe burial of deceased individuals [5]. In contrast, the West African epidemic that began in Guinea in late 2013 and rapidly spread to neighbouring Sierra Leone and Liberia reached an unprecedented scale. According to the World Health Organization (WHO), the outbreak resulted in approximately 30,000 infected individuals and 11,000 deaths before it was controlled in 2016 [6, 7]. The extraordinary magnitude of this epidemic was attributed to several factors, including a delayed diagnosis in a region with no prior history of EBOV outbreaks, and the emergence of the virus in a densely populated area with high levels of cross-border mobility, which facilitated its spread to large urban centres [8].

The EBOV epidemic in West Africa was caused by the Makona variant of EBOV (species *Orthoebolavirus zairense*), which exhibits about 3% divergence from the classic EBOV variants Mayinga 1976 or Kikwit 1995 [9, 10]. Remarkably, evolution of EBOV Makona was closely monitored throughout the epidemic *via* near-complete genome sequencing of viral isolates [8–11]. Although the overall substitution rate of EBOV during the 2013–2016 epidemic was consistent with that observed in previous outbreaks [12, 13], several mutations became fixed across different genomic regions over successive cycles of human-to-human transmission [14–16]. Among these, the substitution A82V in the EBOV glycoprotein (GP) emerged early in the outbreak and was associated with the rapid spread of the virus [14–16]. Notably, this same substitution re-appeared in the 2021 EBOV outbreak in Guinea, which resulted in 16 confirmed cases and 9 deaths. Genomic analysis revealed that EBOV sequences from this outbreak clustered closely with those from the 2013–2016 epidemic, displaying significantly lower divergence than expected. These findings suggest a period of viral latency within the human host, rather than a continuous transmission. These observations highlight the importance of long-term monitoring of Ebola survivors to mitigate the risk of viral re-emergence. Furthermore, continued investigation into the viral and host factors that contributed to the emergence and spread of EBOV Makona remains a critical priority for outbreak preparedness and response [17].

The A82 residue is located near the GP receptor-binding region, and the A82V substitution has been shown to modestly enhance infectivity in human cells compared to other mammals, such as bats [14, 16]. This increased infectivity is attributed to a destabilized GP-V82 structure, which promotes membrane fusion rather than improving binding to the NPC1 receptor [18, 19]. Additionally, GP-V82 exhibits enhanced sensitivity to proteolysis by cathepsin L (CatL) in endosomes, a key host factor for viral entry [18, 20]. The clinical significance of this substitution remains uncertain, as studies in preclinical models (including mice and non-human primates) suggest there are no differences in pathogenicity compared to the Mayinga variant [21]. However, this might be dependent on the route of infection, with evidence suggesting the Makona variant might be less virulent than the Mayinga variant *via* intraperitoneal injection, but potentially more virulent when transmitted *via* aerosol [22].

EBOV cell entry is a complex process involving initial virus attachment to cell surface molecules followed by internalization by macropinocytosis [23]. Once inside endosomes, host proteases cleave the EBOV GP, exposing previously hidden regions that interact with the C domain of the NPC1 receptor [24, 25]. This triggers fusion of the viral and endosomal membranes, leading to the release of the viral genome into the cytoplasm. One of the most well-characterized EBOV attachment factors is dendritic cell-specific ICAM-3 grabbing non-integrin (DC-SIGN, also known as CD209) [26, 27], which specifically recognizes glycosylations on the EBOV GP and other viral glycoproteins. DC-SIGN binds high-mannose and fucosylated Lewis (Le) carbohydrates [28] and is mainly expressed by immature dendritic cells (DCs) in the skin and mucosa, efficiently mediating EBOV entry into both primary and established cell lines [29, 30].

In the present study, we demonstrate that pseudoviruses bearing the GP-V82 variant exhibit enhanced infection of DCs and macrophages compared to those carrying GP-A82. This increased infectivity was inhibited by a DC-SIGN-specific antibody, indicating a key role for this lectin in mediating the enhanced entry. The infection enhancement associated with GP-V82 with or without the mucin-like domain was also recapitulated in DC-SIGN-transduced Jurkat, Raji and K562 cells. The infectivity levels correlated positively with DC-SIGN expression levels. Furthermore, filovirus-like particles (FLPs) composed of EBOV VP40 and GP-V82 displayed faster cell entry kinetics than those containing GP-A82. This enhancement was linked to N-linked glycans at Asn257 and Asn563 on the GP, which we propose facilitate bivalent binding of the DC-SIGN dimer to glycans at these sites [31]. Soluble GP-V82 exhibited higher binding activity to the extracellular domain (ECD) of the DC-SIGN compared to GP-A82. This stronger interaction is likely due to the distinct glycan composition of the GP-V82 variant, as revealed by NMR analysis, which identified an enrichment in the high-affinity Lewis X (Le^x^) epitope. An antibody against Le^x^ (CD15) completely blocked infection by GP-V82 pseudovirus in Jurkat-DC-SIGN cells, whereas only partial inhibition was observed for GP-A82. These findings demonstrate that the increased infectivity of the EBOV Makona GP-V82 variant is closely linked to its differential glycosylation profile.

## Results

### DC-SIGN-dependent enhancement of cell infectivity by the EBOV Makona GP-V82 variant

The GP A82V substitution present in Makona EBOV was initially proposed to facilitate binding to the NPC1 receptor [14, 16]. However, subsequent studies linked the increased infectivity of the Makona GP-V82 to a destabilized GP structure that favours membrane fusion, rather than to improved NPC1 binding affinity [18–20]. Nonetheless, the host factors contributing to the enhanced human cell tropism of the Makona V82 variant remain uncharacterized.

**Figure 1.**
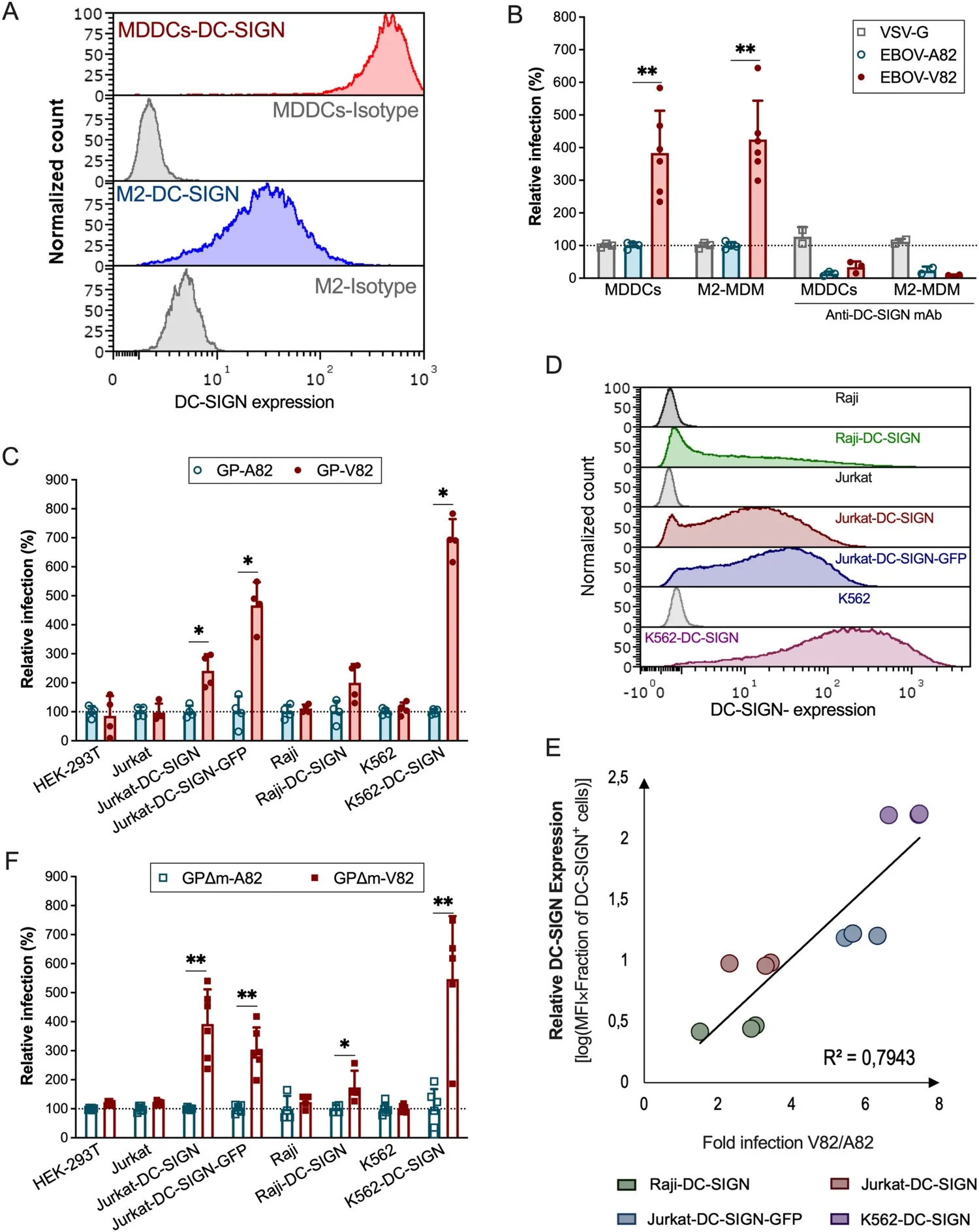
DC-SIGN enhances the infectivity of the EBOV Makona GP-V82 variant. (A) Normalized histograms showing DC-SIGN expression levels in monocyte-derived dendritic cells (MDDCs) and in M2 monocyte-derived macrophages (M2-MDM) measured by flow cytometry with an anti-DC-SIGN PE-labelled antibody (see Methods). Isotype controls are included. (B) DC-SIGN-dependent infection of MDDCs and M2-MDM with pseudovirus containing EBOV GP-A82 or GP-V82. Cells were infected with lentiviruses pseudotyped with the GP or with a control VSV-G protein, and the infectivity determined by luciferase activity at 48 h post-infection (hpi), as described in Methods. Cell infectivity was also measured in the presence of an anti-human DC-SIGN antibody (1621F at 10 μg/ml). Infection (%) relative to GP-A82 in each cell line is plotted for the three pseudoviruses, with and without the DC-SIGN antibody. Cells from three donors were used in three independent experiments performed in triplicates, mean ± SD. (C) Enhanced infectivity of pseudovirus containing GP-V82 in cell lines expressing cell surface DC-SIGN. Jurkat, Raji and K562 cells with or without cell surface DC-SIGN were infected as in B, with HEK-293T as control. Infectivity determined by luciferase activity (RLUs) at 48 hpi is shown in Figure S2, and the relative infection to GP-A82 is plotted here. Mean ± SD of four replicates. (D) DC-SIGN cell expression. Histogram representing DC-SIGN expression in the cell lines used in C, measured as in panel A. (E) Correlation of GP-V82 enhanced cell infectivity with DC-SIGN expression. DC-SIGN expression levels, measured by flow cytometry, and represented as the product of the median fluorescence intensity and the fraction of DC-SIGN^+^ cells, were plotted against the fold increase in GP-V82 infectivity relative to GP-A82 in DC-SIGN-expressing cells. (F) Implication of the mucin-like domain (MLD) in DC-SIGN-dependent infection of pseudovirus with the EBOV GP. Infectivity of the pseudovirus bearing GPΔm-A82 or GPΔm-V82, lacking the MLD, were determined as in C and it is shown in Figure S2; the infection relative to GPΔm-A82 is shown here. Mean ± SD of four to six replicates, from two independent experiments, is represented. For panels B, C and F, comparison between GP-A82 and GP-V82 for each cell line is performed by Mann Whitney U-test. Significance is represented as: *p = 0.05 to 0.01, **p = 0.01 to 0.001.

To explore the role of EBOV receptors, such as the C-type lectin DC-SIGN, we developed a quantitative infectivity assay using EBOV GP-pseudotyped lentiviral particles in monocyte-derived macrophages by M-CSF, broadly characterized as uncommitted M2 macrophages without further polarization (from now on, M2-MDMs) and in monocyte-derived dendritic cells (MDDCs). These primary human cell models express significant levels of surface DC-SIGN, with MDDCs showing a higher expression than M2-MDMs (Figure 1(A)) [32]. In these assays, pseudoviruses bearing GP-V82 infected M2-MDMs and MDDCs about four times more efficiently than those bearing GP-A82 (Figure 1(B)). Lentiviral particles bearing the GP from vesicular stomatitis virus (VSV-G) were used as an infection control. Notably, infection of both MDDCs and M2-MDMs was inhibited by a DC-SIGN specific antibody, indicating that a DC-SIGN-dependent mechanism contributes to the enhanced entry of the Makona GP-V82 variant (Figure 1(B)).

To further investigate the role of DC-SIGN in EBOV Makona infection, we used a CD4^+^ Jurkat T cell line modified *via* retroviral transduction to express DC-SIGN (Jurkat-DC-SIGN) or DC-SIGN fused to GFP (Jurkat-DC-SIGN-GFP) [29]. Similarly, we transduced a Raji B cell line and lymphoblast K562 cell line to express DC-SIGN, generating Raji-DC-SIGN and K562-DC-SIGN, respectively [33]. T-lymphocytes such as Jurkat cells and B-lymphocytes are naturally non-permissive to EBOV infection, while K562 cells exhibit low susceptibility [29, 34]. Nonetheless, expression of DC-SIGN alone was sufficient to allow or increase entry and infection by EBOV Makona GP-pseudotyped lentiviruses in all cell lines (Figure 1(C)). Furthermore, DC-SIGN expression significantly enhanced infection by the GP-V82 compared to the GP-A82 variant, with up to 7-fold in K562-DC-SIGN cells (Figure 1(C)). Flow cytometry analysis of DC-SIGN expression revealed distinct differences between non-transduced and transduced cell lines, with no detectable DC-SIGN expression in non-transduced cells and variability among transduced cells in both the proportion of DC-SIGN-positive cells and the level of expression per cell (Figure 1(D)). Among the lines tested, Raji-DC-SIGN cells exhibited the lowest expression, followed by Jurkat-DC-SIGN, Jurkat-DC-SIGN-GFP, and K562-DC-SIGN, which showed the highest levels. Remarkably, a strong positive correlation (R^2^ = 0.7943) was observed between DC-SIGN expression level and the fold increase of the GP-V82 infectivity relative to GP-A82 (Figure 1(E)), further supporting a direct role for DC-SIGN in mediating the enhanced entry of the Makona V82 variant.

### DC-SIGN-mediated enhancement of EBOV GP-V82 pseudovirus infection is independent of the mucin-like domain

The mucin-like domain (MLD) of EBOV GP is a heavily glycosylated domain, and its structure is undefined [36]. Additionally, while N-linked glycosylation sites in other domains (base, head and glycan cap) are relatively well conserved among *orthoebolavirus* species, those in the MLD are more variable (Figure S1) [31]. Thus, we aimed to investigate DC-SIGN-dependent enhancement of infection with pseudovirus bearing GP without the MLD. Removal of the MLD in the GP (GPΔm) resulted in pseudoviruses more infectious than those with the full-length GP in cells without DC-SIGN (Figure S2), as described previously for EBOV by other authors [37]. Nonetheless, expression of DC-SIGN in Raji, Jurkat and K562 cells further increased the infectivity of GPΔm-V82 compared to GPΔm-A82 (2 to 7-fold depending on the cell line) (Figure 1(F)), analogous to the results observed with pseudovirus bearing the full-length GP (Figure 1(C)). Consequently, we concluded that the infectivity enhancement of the GP-V82 variant was DC-SIGN dependent and MLD-independent.

### Enhanced DC-SIGN-dependent infection by EBOV GP-V82 pseudovirus produced in K562 cells

N-glycosylation of the GP could greatly vary depending on the cell line used to produce pseudoviruses, which in turn could affect DC-SIGN-dependent enhancement of infectivity [35]. To assess this, we prepared pseudotypes bearing GP-A82 and GP-V82 in K562, a hematopoietic cell line quite distinct from HEK-293T cells. They had an analogous behaviour to those produced in HEK-293T cells. Although the virus titres were overall lower when particles were produced in K562 cells than in HEK-293T cells, they showed comparable infectivity in HEK-293T cells (Figure S3(A)). In Jurkat, very low infection was observed in the absence of DC-SIGN (Figure S3(A)), whereas a significant DC-SIGN-dependent enhancement of GP-V82 infectivity was detected in Jurkat expressing DC-SIGN compared with GP-A82 (Figure S3(A,B)). In terms of relative infection, GP-V82 showed about 6-fold increase relative to GP-A82. This finding is consistent with the enhanced DC-SIGN-dependent infection of GP-V82 shown above with pseudoviruses produced in other cell lines.

### Accelerated cell entry of filovirus-like particles (FLPs) bearing the Makona GP-V82

**Figure 2.**
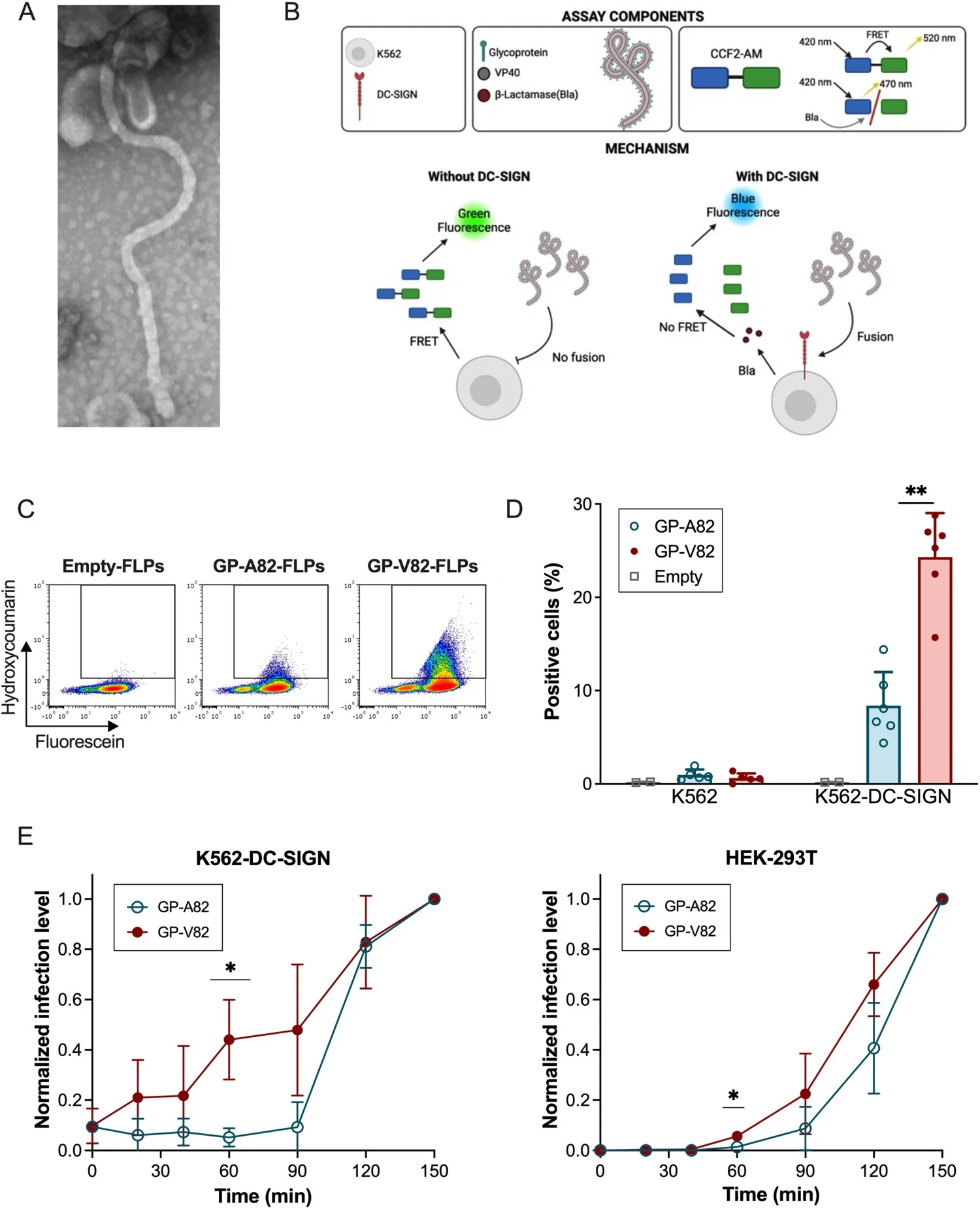
Faster cell entry of filovirus-like particles with the Makona GP-V82 envelope. (A) Representative electron microscopy image of a filovirus-like particle (FLP). (B) FLP cell entry assay. FLPs containing EBOV-GP (A82 or V82), EBOV VP40 and β-lactamase (Bla) successful fusion induces change in fluorescence from green to blue in the presence of CCF2-AM2, which can be measured by flow cytometry. Figure created with Biorender.com. (C) Flow cytometry measuring infection of K562-DC-SIGN cells with GP-VP40-Bla FLPs. Positive infected cells are boxed. Assay described in Methods. (D) FLP infection of cells with and without DC-SIGN. The percentage of positive, infected cells was determined as in (C) with K562 or K562-DC-SIGN cells after 1 h of spinoculation with 10 μg of FLPs at 4°C and after 3 h of incubation at 37°C. Comparison between GP-A82 and GP-V82 is performed by Mann Whitney U-test. Mean ± SD from six replicates, from two independent experiments are presented. Significance is represented as: **p = 0.01 to 0.001. (E) Kinetics for FLP entry into cells with and without DC-SIGN. FLP cell entry and infection was stopped by the addition of chloroquine (100 μM) at the indicated time points. The percentage of infected K562-DC-SIGN or HEK-293T cells were determined at different incubation times as in (D), and the normalized infection level calculated as the ratio of infected cells relative to the total cells infected 150 min post-incubation with the GP-A82 or GP-V82 FLPs. Mean ± SD of three replicates per timepoint, FLP and cell line. Statistical comparisons were performed via two-way repeated-measures ANOVA with Geisser-Greenhouse correction, followed by Fisher’s LSD multiple-comparisons test. Significance is presented as: *p = 0.05.

To account for potential effects of the lentiviral pseudotyping system in the EBOV-GP infectivity, we generated FLPs that mimic authentic filoviral morphology. These FLPs were composed of EBOV GP (either A82 or V82), EBOV-VP40 and β-lactamase (Bla) (EBOV-GP-VP40-Bla, representative image shown in Figure 2(A)). Morphologies of the FLPs ranged from elongated filamentous forms to smaller spherical particles, with no obvious differences observed between FLPs bearing GP-A82 or GP-V82. Control FLPs lacking GP (empty particles) were also included. No marked differences in the incorporation of GP-A82 versus GP-V82 into FLPs were detected (Figure S4). To monitor FLP cell entry, we performed an assay that includes the use of the CCF2-AM reagent (see Methods), so that the successful fusion of FLPs with the target cell triggered a fluorescence shift from green (fluorescein) to blue (hydroxycoumarin) (Figure 2(B)), which was quantified by flow cytometry (Figure 2(C)). Notably, the FLPs bearing the GP-V82 variant exhibited about a 3-fold increase in cell entry compared to those with GP-A82, but this enhancement was observed exclusively in K562 cells expressing DC-SIGN (K562-DC-SIGN cells) (Figure 2(C,D)).

This FLP-based system also enabled a reliable study of early events in EBOV cell entry. While macropinocytotic uptake of EBOV occurs relatively quickly, it has been shown that viral RNA enters the cytoplasm only after a considerable delay compared to other viruses [38]. Specifically, EBOV requires trafficking to late endolysosomal compartments enriched in NPC1 and with elevated CatL activity before cytoplasmic entry. These findings suggest that EBOV must move deep into the endocytic pathway to access the proteolytic environment necessary for membrane fusion and genome release. Interestingly, the GP-V82 variant has been reported to exhibit increased sensitivity to CatL-mediated proteolysis in the endosome [20], potentially allowing it to fuse and release its genome earlier in the endocytic pathway. To explore the role of DC-SIGN in modulating EBOV cell entry kinetics, we infected HEK-293T cells and K562-DC-SIGN cells with EBOV FLPs and inhibited acid-dependent endosomal enzymes with hydroxychloroquine at various time points. In K562-DC-SIGN cells, FLPs bearing GP-A82 displayed a delayed entry of about 90 min. This was not observed with GP-V82 FLPs, which showed continuous and accelerated entry kinetics (Figure 2(E)). Two-way repeated-measures ANOVA revealed a significant time × group interaction (*p* = 0.0138), indicating differential kinetics between GP-A82 and GP-V82. In HEK-293T cells lacking DC-SIGN, FLPs with either GP-A82 or GP-V82 exhibited similar entry kinetics, although GP-V82 showed slightly faster entry than GP-A82 (Figure 2(E)), perhaps due to the lower structural stability and higher sensitivity to endosomal factors that promote fusion. Differences in HEK-293T cells were also significant for the time × group interaction (*p* = 0.0298). Specifically, differences between GP-A82 and GP-V82 were significant 60 min postinfection, for both K562-DC-SIGN cells (*p* = 0.0452) and HEK-293T cells (*p* = 0.0295). Nonetheless, the presence of DC-SIGN on the cell surface markedly influenced the entry dynamics, eliminating the delay for GP-V82-containing FLPs and facilitating more rapid cytoplasmic access (Figure 2(E)).

As the morphologies of the FLPs ranged from filamentous forms to smaller spherical particles, differences in entry efficiency between GP-A82 and GP-V82 FLPs could be driven by small spherical particles that can co-purify in standard FLP preparations. To assess this possibility, we enriched our preparations for the longer filamentous FLPs using a discontinuous sucrose step gradient (10%, 30%, and 60%) after the initial 20% sucrose cushion purification. This is the standard protocol used for purifying filamentous particles [39, 40]. Fractionation of the FLP preparations confirmed the co-migration of most GP1 and VP40-Bla into the gradient fractions corresponding to the 60 and 30% sucrose interphase (Fractions F2-F4), consistent with the sedimentation of filamentous particles (Figure S5(A–C)), and confirmed by electron microscopy. Fractionated particles bearing GP-V82 exhibited higher infectivity in K562-DC-SIGN cells than those bearing GP-A82 (Figure S5(D)), similar to the pattern observed with unfractionated FLPs (Figure 2(D)). These data suggest that the entry advantage conferred by the A82V substitution in DC-SIGN-expressing cells is retained in filamentous particles that mimic the morphology and uptake mechanisms of wild-type filoviruses.

### EBOV Makona GP-V82 exhibits higher DC-SIGN binding activity than GP-A82

**Figure 3.**
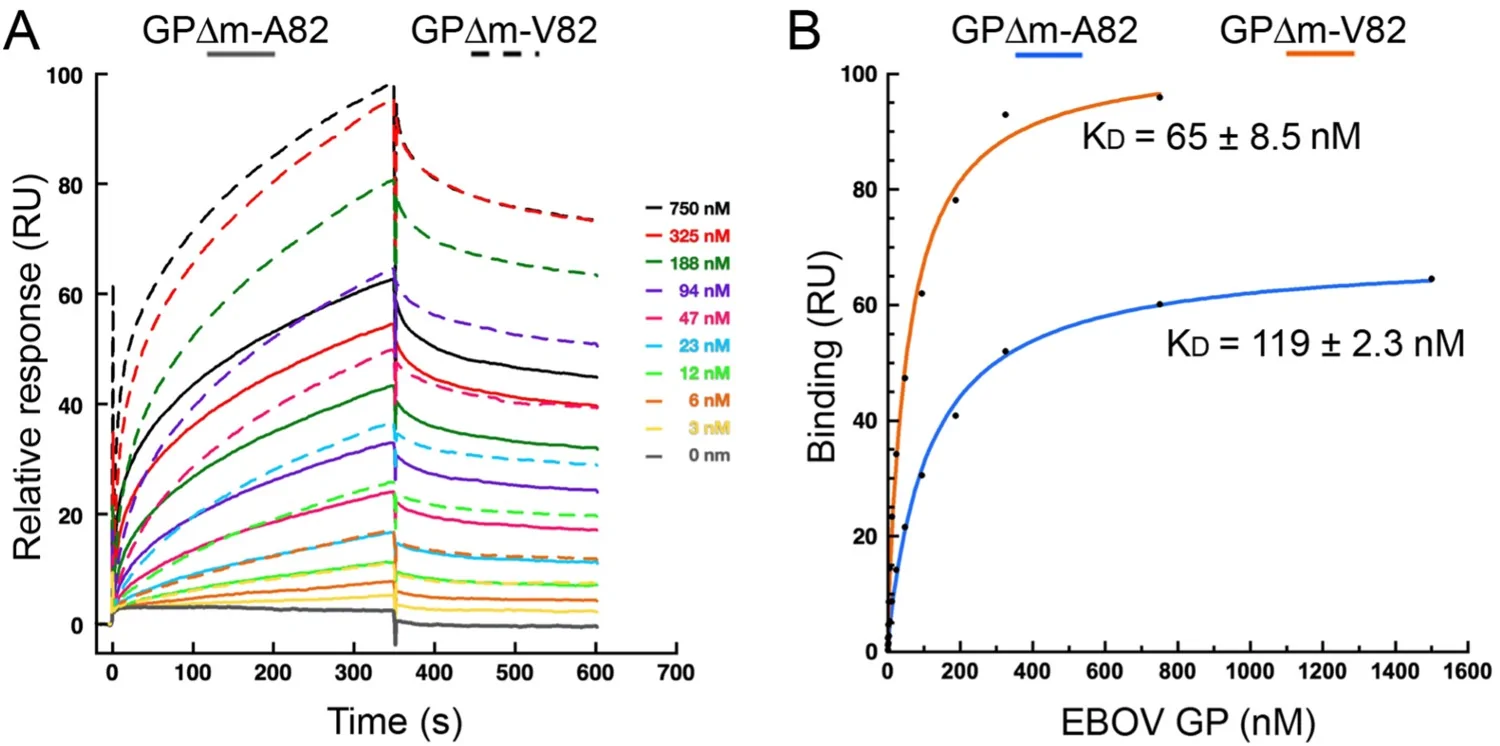
Interaction of soluble EBOV GP variants with a DC-SIGN oriented surface by SPR. (A) Overlayed sensorgrams for the real time binding of EBOV GPΔm-A82 and GPΔm-V82 variants without the MLD to DC-SIGN ECD oriented surfaces. Sensorgrams were recorded for the indicated GP protein concentrations as described in Methods. (B) Binding of the GP variants to DC-SIGN computed from the sensorgrams. The determined apparent KD for the two GP variants, as described in Methods, is shown. Mean ± SD of 3 independent titration experiments.

After demonstrating that EBOV GP-V82 has an enhanced infectivity *via* DC-SIGN, and that this effect was independent of the MLD, we compared the binding of soluble GPΔm-A82 and GPΔm-V82 proteins to the ECD of DC-SIGN using surface plasmon resonance (SPR). To mimic the native presentation of the receptor on the cell surface, a DC-SIGN ECD with a N-terminal biotinylation was directionally immobilized on a sensor chip (see Methods), and varying concentrations of GPΔm-A82 or GPΔm-V82 were injected over the same surfaces (Figure 3(A)). GPΔm-V82 consistently showed stronger binding to DC-SIGN than GPΔm-A82 (Figure 3(B)), with approximately 2-fold higher apparent affinity based on the response units (RU) measured at the end of the injection phase. Moreover, a higher absolute level of binding can be observed on the same surface with the GPΔm-V82 with respect to the GPΔm-A82 (Figure 3(B)). This difference at the level of soluble GP molecules could be further amplified by the engagement of multiple GPs on the viral surface, thereby enhancing virus attachment to the cell-surface receptor DC-SIGN and resulting in the increased infectivity observed for lentiviruses and FLPs (Figures 1 and 2). Moreover, the observation that a single GP trimer interacts with a native-like DC-SIGN ECD surface in the nM range suggests that the overall binding strength of the virus to the cell could be substantially higher due to avidity effects from multiple interactions involving several GP trimers on the viral envelope.

### N-linked glycans in the Makona GP that enhance the V82 variant infectivity via dimeric GP binding to DC-SIGN

**Figure 4.**
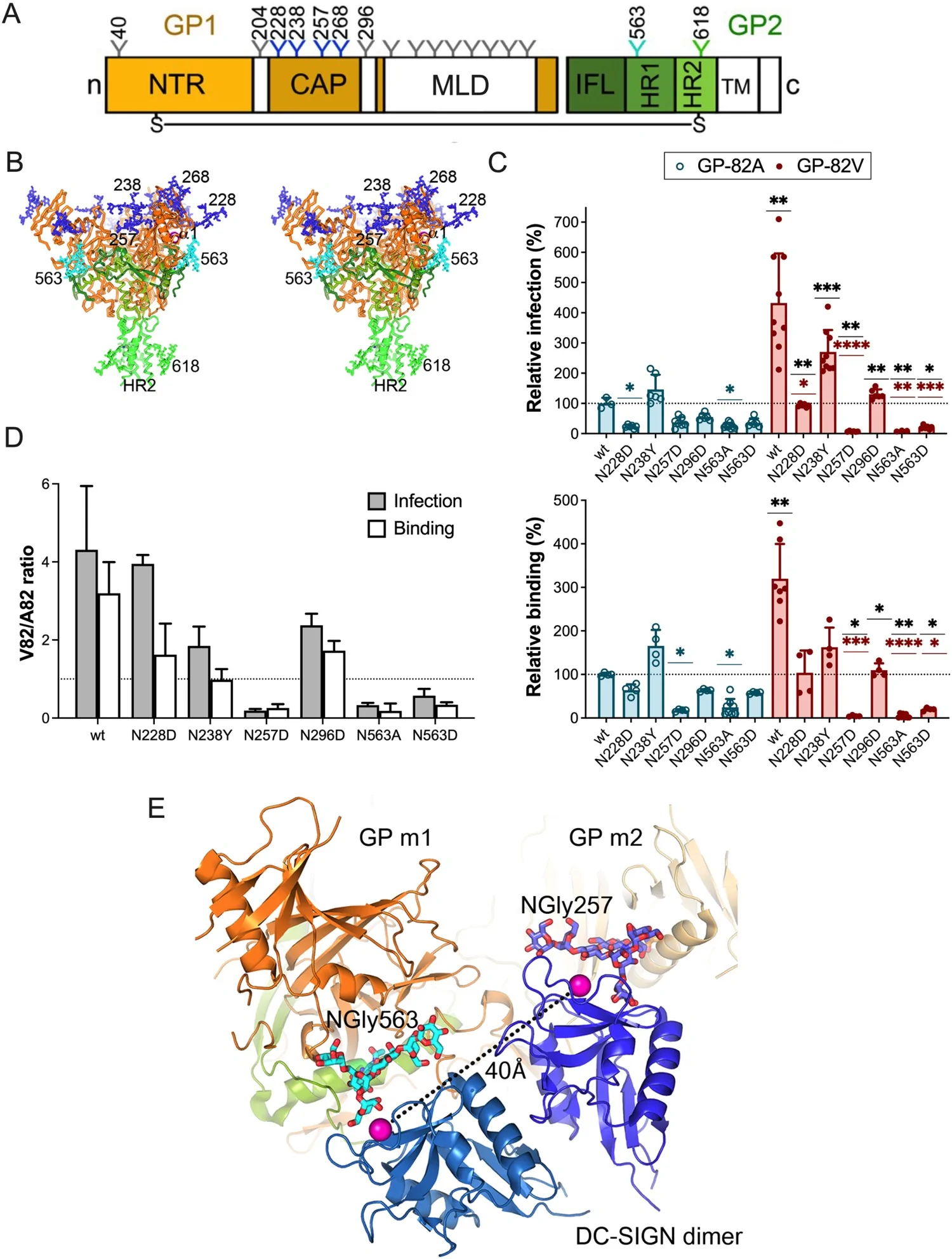
N-linked glycans in the EBOV GP that enhance the V82 variant infectivity and GP/DC-SIGN binding model. (A) N-linked glycosylation in the EBOV GP. Scheme of the GP1 and GP2 regions in orange and green, respectively, and the absent domains in reported crystal structures (mucin, transmembrane and cytoplasmic) in white. N-terminal region (NTR), glycan cap (CAP), internal fusion loop (IFL), and heptad repeat (HR) regions defined in GP structures are coloured, with N-linked glycosylation sites as Y; undefined sites in the structures are shown in grey and those in the MLD not numbered (see Figure S1). (B) Stereo view of a trimeric EBOV GP structure (PDB ID 5JQ3) with modelled Man-5 glycans in the defined N-linked glycosylation sites (see Methods). One monomer is shown as ribbons with labelled glycans, and the other two as Cα representations. The GP1, GP2 regions and N-linked glycans are coloured as in the panel A scheme. The alpha helix 1 (α1) in GP1 that bears the A82V substitution is in magenta (shown also in Figure S6). (C) Identification of N-linked glycans responsible for enhanced GP-V82 cell binding and infectivity. DC-SIGN-dependent binding (bottom) or infectivity (top) of lentiviral particles pseudotyped with wild type GP or N-linked glycosylation mutants, with the indicated substitution at the Asn residues. DC-SIGN-dependent binding and infection of Jurkat-DC-SIGN cells were determined for each mutant after subtraction of values obtained in parental Jurkat cells, and the relative values determined with respect to the GP-A82 wt (see Methods). Individual dots represent individual replicates. Mean ± SD. Comparison between GP-A82 and GP-V82 for each mutant was performed by Mann Whitney U-test. Significance is represented over each GP-V82 mutant in black. Comparisons between the mutants and the corresponding wild-type GP for each variant were performed using the Kruskal-Wallis procedure, followed by the Dunn multiple comparison test; the results are shown above each mutant, in blue for GP-A82 and in red for GP-V82 mutants. Significance levels are indicated as: *p = 0.05 to 0.01, **p = 0.01 to 0.001, ***p = 0.001 to 0.0001. (D) GP-V82 binding and infectivity with respect to GP-A82 in the glycosylation mutants. V82/A82 ratios were determined for the WT and each mutant from the data in C. (E) Simultaneous engagement of the EBOV GP Ngly257 and Ngly563 with the ECD of DC-SIGN. Model of the putative interaction of the EBOV GP with the two glycans that are essential for the DC-SIGN-dependent GP-V82 enhanced infection. Two contiguous monomers (bright and light orange) of the GP trimer with modelled Man-5 glycans as in panel B are bound to a DC-SIGN dimer (half of the native DC-SIGN tetramer not entirely presented here for clarity). Ribbon representation of the lectin dimer (shown in blue) is based on the DC-SIGNR crystal structure (PDB ID 1XAR); the carbohydrate-binding calcium ions at about 40 Å in the dimer are shown as magenta spheres. The preparation of the GP-bound lectin dimer is described in Methods.

DC-SIGN preferentially recognizes high-mannose or fucosylated Lewis-type glycans that are commonly N-linked to viral glycoproteins, such as the EBOV GP [41], which contains 17 N-linked glycosylation sites, 15 of which are located in the GP1 subunit (Figure 4(A) and Figure S1). These sites cluster within the membrane-distal glycan cap and the mucin-like domain, the latter being absent in available structures (Figure 4(B)). The glycan cap with four N-linked glycosylations overlays the receptor-binding region and the alpha helix 1 (α1) that harbours the A82V substitution (Figure 4(B) and Figure S6). This helix is further shielded by a glycan N-linked to Asn563 (Ngly563) in the HR1 motif of GP2 (Figure S6). Ngly563 is conserved across filovirus GPs (Figure S1), and it is well-resolved in several GP crystal structures [42–44] due to stabilizing contacts with the protein that limit its flexibility (Figure S6). In contrast, the other N-linked glycans in GP exhibit high inherent flexibility in the crystal structures [42–44]; Asn204 and Asn296 glycosylation sites are located in unresolved regions at the N- and C-termini of the GP1 glycan cap, respectively (Figure 4(A)). These flexible glycans likely contribute to EBOV immune evasion by hindering antibody access. It has been reported that individual mutations of GP1 glycans do not affect GP processing or functionality [45]. In contrast, deletion of Ngly563 or Ngly618 in GP2 can impact GP processing, particularly in the absence of the MLD; however, these glycans are not necessary for EBOV transduction in HEK-293T cells [45].

To identify the specific N-linked glycosylations responsible for the enhanced infectivity of the Makona GP-V82 variant, we performed deletion of individual N-linked glycosylation sites near α1. All glycosylation mutants were successfully incorporated into pseudotyped lentiviruses, as measured by western blot (Figure S7). Removal of GP1 glycans Ngly228, Ngly257 and Ngly296, as well as Ngly563 from GP2 reduced Jurkat-DC-SIGN cell binding and infectivity of both GP-A82 and GP-V82 pseudotyped lentiviruses by over 50%; deletion of Ngly238 had minimal effect on GP-A82, but reduced GP-V82 infectivity (Figure 4(C)). Ngly228 was most critical for DC-SIGN-dependent Jurkat cell infection of the GP-A82 variant, whereas Ngly257 and Ngly563 were essential for the binding and infectivity of the GP-V82 variant (Figure 4(C)). Notably, only deletion of Ngly257 or Ngly563 abolished the increased DC-SIGN-mediated infection conferred by the V82 substitution, resulting in lower infectivity of GP-V82 compared to GP-A82 (Figure 4(D)). In contrast, deleting the other glycans maintained the V82-related benefits. These findings identify Ngly257 and Ngly563 as key determinants of the increased DC-SIGN-dependent infectivity of the EBOV GP-V82 variant, consistent with previous reports suggesting these N-linked glycans as potential DC-SIGN ligands based on their glycosylation composition [31].

Ngly257 and Ngly563, the two glycans responsible for the enhanced infectivity of the EBOV Makona GP-V82 variant, are distant both in the primary sequence and the GP monomer structure; however, they are brought into close spatial proximity in the trimeric GP conformation (Figure 4(B)). The distance between their β-mannose residues (∼40 Å) closely matches the separation between the carbohydrate-binding sites of two DC-SIGN monomers within a lectin tetramer (Figure 4(E)). Structural superposition of the carbohydrate recognition domains (CRDs) of DC-SIGN bound to carbohydrates with Ngly257 and Ngly563 demonstrated that a DC-SIGN dimer can simultaneously engage both glycans (Figure 4(E)). The dual engagement likely underlies the enhanced binding and infectivity observed with the GP-V82 variant, as supported by the loss of this enhancement in single glycosylation mutants (Figure 4(C,D)). This dimeric interaction is even more plausible if we consider the flexibility and adaptability of the individual carbohydrate recognition domains of DC-SIGN within the tetramer. This has been documented recently and makes it possible to optimize multivalent binding to different target types and thus generate these avidity properties [46].

### Differential glycosylation content in the Makona GP-A82 and GP-V82 proteins

Recent studies have shown that the N-gly257 and N-gly563 are enriched in oligomannose and hybrid-type glycans, particularly in the N-gly563, which contains less than 10% complex glycans when expressed in HEK-293 cells [31]. This glycosylation profile makes both sites well-suited for recognition by DC-SIGN. Nonetheless, the glycan composition of the GP-A82 and GP-V82 proteins has not been compared. We addressed this by NMR spectroscopy on soluble proteins lacking the mucin domain, produced in FreeStyle 293-F cells (ThermoFisher) and labelled with [^13^C]-glucose, as described in Methods. This labelling strategy enables selective ^13^C-labelling of glycans with minimal amino acid scrambling. Under these conditions, high quality ^1^H,^13^C-HSQC NMR spectra can be acquired without sensitivity limitations [47, 48]. Each glycan produces distinct signals in the HSQC spectrum, providing a unique glycosylation fingerprint for the protein, as demonstrated in various systems [49, 50].

**Figure 5.**
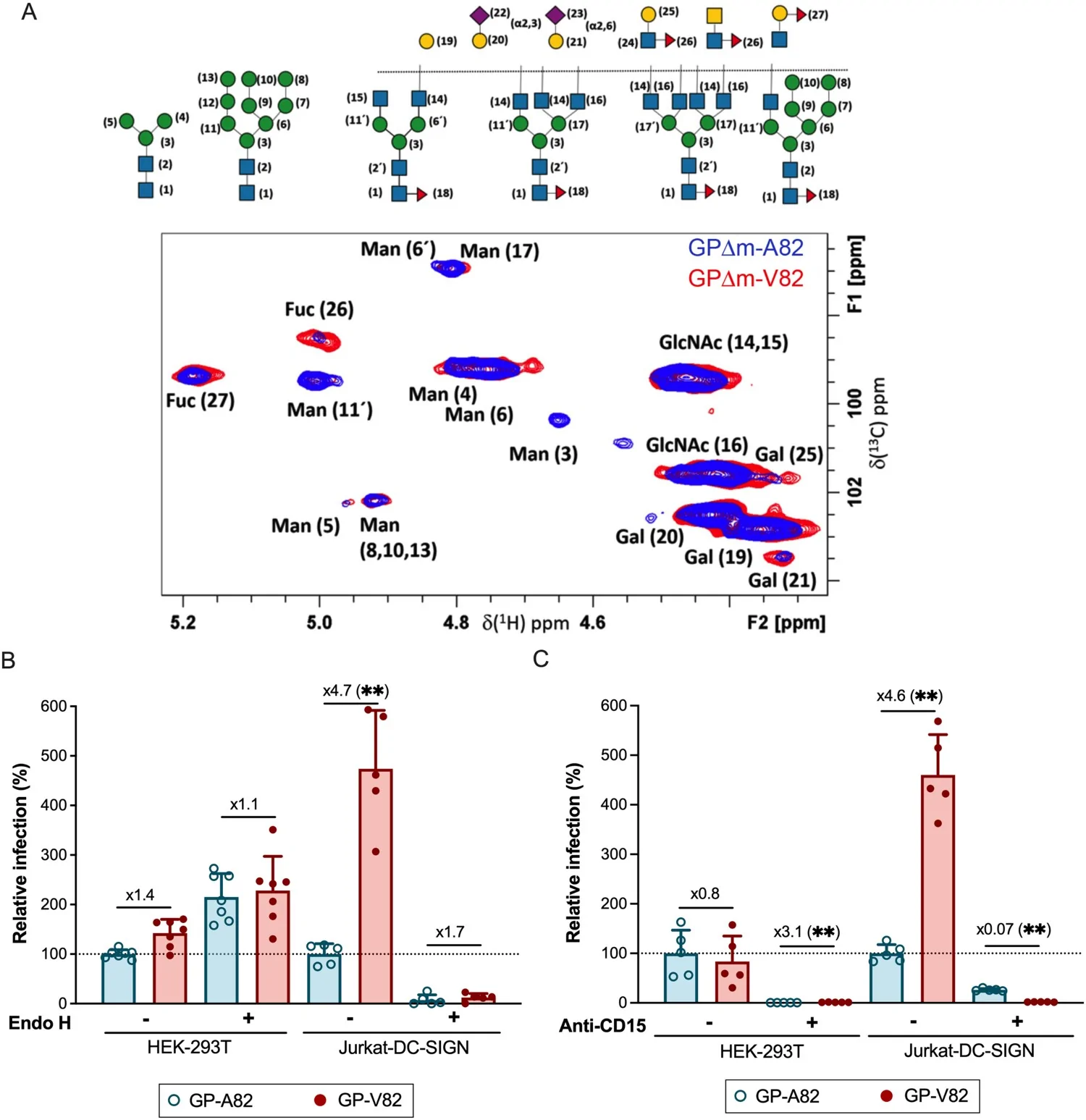
Differential glycan composition in the EBOV GP-A82 and GP-V82 variants and contribution of the glycans to the infectivity of the EBOV GP variants. (A) NMR identification of glycan structures in the GP variants. Expansion of the anomeric region of ^1^H-^13^C HSQC spectra showing (C1-H1) correlations. GPΔm-A82 spectrum (blue colour) is overlaid with the recorded spectrum of GPΔm-V82 (red colour), as described in Methods. The identified glycans in the spectrum are represented as SNFG symbols on the top [72]. (B, C) Probing the role of high-mannose and Le^x^ glycan ligands of DC-SIGN in enhancing GP-V82 infectivity. (B) Impact of endo H treatment on EBOV GP-mediated lentivirus infectivity. Relative infection (%) of the endo H-treated (+) or untreated (-) lentiviruses bearing GP-A82 or GP-V82 in HEK293T or Jurkat DC-SIGN cells was determined with respect to the GP-A82 sample in HEK293T cells or in Jurkat DC-SIGN, respectively. Fold-change in infection between the GP variants is indicated. (C) Evaluating the effect of an anti-CD15 (Le^x^) antibody in GP-mediated lentivirus infectivity. Relative infection (%) of the pseudovirus bearing GP-A82 or GP-V82 and incubated with a saturating concentration (see Figure S9) of anti-CD15 (Le^x^) antibody (+) or without antibody (–) in HEK293T or Jurkat DC-SIGN, determined as in (B). In (B) and (C) each biological replicate is individually represented as an individual dot, with bar height representing the mean and error bars the SD. Fold increase of GP-V82 over GP-A82 is shown. Comparison between GP-A82 and GP-V82 for each condition was performed by Mann Whitney U-test. Significance is included in parenthesis as: *p = 0.05 to 0.01, **p = 0.01 to 0.001.

The ^1^H,^13^C-HSQC NMR spectrum of GPΔm-A82 (Figure 5(A), blue) revealed signals corresponding to mannose (Man), N-acetylglucosamine (GlcNAc), galactose (Gal) and fucose (Fuc), indicating the presence of diverse glycan structures. Characteristic peaks for high-mannose glycans, such as those labelled as Man 8,10,13 in Figure 5(A), were identified, confirming structural motifs known to interact with DC-SIGN [28]. Additionally, the signals consistent with complex-type N-glycans (bi-, tri- and tetra-antennary structures) and hybrid glycans with varying degrees of sialylation were observed. Fucose α(1-3)-linked to GlcNAc was detected, indicating the presence of Le^x^ structures. Signals for sialic acids linked to galactose via α(2-3) or α(2-6) bonds were identified as well (Figure S8).

The superimposed spectrum of the GPΔm-V82 (Figure 5(A), red) showed a similar overall glycan profile but with increased intensity of peaks marked as Fuc 26 and Gal 25, components of the Le^x^ structural motif, a high affinity DC-SIGN ligand [28, 51, 52]. These data suggested a preferential enrichment of Le^x^ in the GPΔm-V82 variant. This enrichment may account for the increased DC-SIGN binding activity observed for GPΔm-V82 compared to GPΔm-A82, likely due to a higher density of high-affinity lectin ligands in the V82 variant.

### Probing the contribution of DC-SIGN ligands in the infectivity of the EBOV GP variants

NMR analysis confirmed the presence of glycosylations on the GP that serve as ligands for DC-SIGN, potentially contributing to the enhanced DC-SIGN-dependent infectivity of the GP-V82 variant (Figure 5(A)). To investigate the role of N-linked high-mannose glycans in this process, we treated GP-A82 and GP-V82 pseudotyped lentivirus with endoglycosidase H, which selectively removes high-mannose glycans, prior to infection of HEK-293T or Jurkat cells stably expressing DC-SIGN. In HEK-293T cells, GP-V82 did not show increased infectivity compared to GP-A82 (Figure 5(B)) and endo H treatment resulted in a modest increase in infectivity of GP-A82 (∼2-fold) and GP-V82 (∼1.5-fold) pseudoviruses relative to untreated controls (Figure 5(B)). In contrast, endo H treatment abrogated infection of Jurkat DC-SIGN cells, and GP-V82 was not significantly more infective than GP-A82 in Jurkat DC-SIGN cells (Figure 5(B)). These findings showed a critical role of high-mannose glycans in mediating DC-SIGN-dependent entry of lentiviruses bearing EBOV Makona GP.

Subsequently, we investigated the role of Le^x^, a glycan structure identified on the GP by NMR analysis. Pseudotyped lentivirus bearing either GP-A82 or GP-V82 was pre-incubated with an anti-Le^x^ antibody (mouse anti-human CD15, Clone MMA) prior to infection of HEK-293T or Jurkat-DC-SIGN cells. Notably, high concentrations of the antibody (10 μg/mL) effectively blocked the infection of HEK-293T cells, achieving 99.4% and 98.8% neutralization for GP-A82 and GP-V82, respectively (Figure 5(C)). This could indicate blocking of the interaction between the virus and alternative glycan-binding molecules (other than DC-SIGN) in the surface of HEK-293T cells [53, 54]. Regardless, this phenomenon was not affected by the A82V substitution. In contrast, infection of Jurkat-DC-SIGN cells revealed a differential sensitivity. The anti-Le^x^ antibody inhibited GP-V82 pseudovirus infection by 98.1%, whereas the GP-A82 pseudovirus was only partially inhibited (73.7%) at saturating antibody concentrations (Figure 5(C) and Figure S9). These findings suggest that the interaction of the GP-V82 pseudovirus with DC-SIGN is more reliant on Le^x^ glycosylation compared to GP-A82, consistent with the higher abundance of this glycan in GP-V82 as indicated by NMR analysis (Figure 5(A)). This elevated Le^x^ content likely contributes to the enhanced DC-SIGN-mediated binding and infectivity observed for the Makona GP-V82 variant in this study.

## Discussion

The scale of the 2013-2016 West African *Ebola Virus* Disease epidemic prompted speculation about potential changes in EBOV or its infectious mechanisms that could account for its unprecedented spread [55]. For the first time, extensive sequential human-to-human transmissions of EBOV occurred, potentially favouring the selection of adaptive mutations to humans. One such mutation, the EBOV Makona GP A82V substitution, emerged early in the outbreak and became dominant in circulating strains [14, 16]. Biochemical analysis revealed that the A82V substitution in the GP increased its fusogenic activity and accelerated processing by CatL [18, 20], suggesting a mechanism for enhanced infectivity. However, the documented case-fatality rate during the epidemic remained comparable to previous outbreaks [56], and animal models studies did not support increased pathogenicity associated with the GP A82V substitution [21, 57].

Besides A82V, other EBOV GP mutations have emerged that may improve the virus adaptation to the host. Most notably, V75A, that emerged during the 2018–2020 EBOV epidemic, has been recently shown to increase viral infectivity in both cell lines and murine models, through an increased binding affinity to NPC1 and reduced cysteine protease dependence in the endosome for viral entry [58]. Mutation T544I, observed during various EBOV outbreaks, has been linked to altered cell tropism in non-human primate-derived cells and certain human cells. However, it has also been hypothesized that the mutation might be a tissue culture adaptation, that is exclusive of *Zaire ebolavirus* species [59, 60]. In contrast to the mechanisms previously reported, which primarily affect the interaction with the endosomal receptor NPC1 or the protease processing, the adaptation we describe here affects the initial attachment to the human lectin DC-SIGN, a distinct viral entry stage.

Using lentiviral vectors pseudotyped with the Makona GP of EBOV, we observed enhanced cell binding and infectivity in primary dendritic cells (DCs) and macrophages by the GP-V82 variant in a DC-SIGN-dependent manner. This enhancement was also observed in DC-SIGN-transduced cell lines, where infection levels correlated with DC-SIGN expression. However, this correlation should be interpreted with caution, as additional differences among these cell lines may also contribute to the observed trend. Notably, the DC-SIGN-dependent relative enhancement of the GP-V82 variant infection when compared to GP-A82 occurred independently of the cell type used for pseudovirus generation and remained evident even after removal of the MLD from GP. These findings were confirmed using filoviral-like particles, which more closely mimic the structural presentation of GP on authentic virions. The confirmation of the GP-V82 phenotypic advantage across structurally distinct platforms (both spherical lentiviruses and pleomorphic FLPs) suggests that the molecular mechanism is independent of the macro-scale particle geometry or length distribution. Collectively, these results demonstrated that the A82V substitution in the Makona GP facilitates cell entry and infection via DC-SIGN, identifying this lectin as a host factor that likely contributed to the increased human-to-human transmission and epidemiological success of the EBOV Makona GP-V82 variant during the 2013 outbreak. However, our results also suggest that multiple mechanisms may contribute to the more efficient cell entry of GP-V82. FLP experiments revealed delayed entry kinetics for GP-A82 in DC-SIGN-expressing cells and, to a lesser extent, in cells lacking DC-SIGN, likely reflecting differences at multiple stages of the entry process rather than a single underlying cause. The approximately 90-minute delay observed for GP-A82 may stem in part from less efficient DC-SIGN-mediated attachment, as discussed throughout the manuscript, but could also involve altered trafficking through endosomal compartments. Specifically, GP-A82 may require progression to later, more protease-rich endosomes before productive fusion can occur, consistent with its reduced susceptibility to CatL processing and lower fusogenic activity compared to GP-V82 [18–20].

We demonstrated that the enhancement of GP-V82 infectivity by DC-SIGN is glycosylation-dependent and identified two critical N-linked glycans at positions Asn257 and Asn563 of the GP as essential for efficient cell infection. Interestingly, these two glycosylation sites have been reported to carry high levels of unprocessed glycans, including oligo-mannose and hybrid-type carbohydrates, which are recognized by the DC-SIGN lectin [31]. In this study, we confirmed that high-mannose, particularly abundant in the Ngly563 [31], are essential for efficient infection of both EBOV Makona GP-A82 and GP-V82. Elimination of either N-linked glycosylation site in the GP-V82 variant impaired DC-SIGN binding, suggesting that simultaneous engagement of the lectin is necessary for stable attachment, as illustrated in Figure 4(E). Given that DC-SIGN forms tetramers on the cell surface [61], it is plausible that two distinct GP trimers on the viral envelope engage a single DC-SIGN tetramer, facilitating multivalent binding.

Interestingly, SPR analysis revealed that GP-V82 exhibits higher binding activity to DC-SIGN compared to GP-A82, implying potential differences in glycosylation profiles between the two variants. This hypothesis was supported by NMR analysis, which confirmed altered glycan composition in GP-V82. This could be related to the reduced thermal stability and increased flexibility of this variant, which may result in differential GP processing in ERGIC compartments. Consistently, based on molecular dynamics simulations, Durham et al. reported that GP-V82 exhibits increased conformational dynamics compared with GP-A82 [19], particularly in the GP1 alpha helix1 with the A82V mutation, the GP2 fusion loop, and the N-terminal portion of the HR1 helix that contains Asn563 (see Figure S1). The enhanced flexibility of GP-V82 may increase the accessibility of glycosylation sites to glycosyltransferases, especially for Ngly563. This may result in distinct glycan processing, leading to differences in the glycosylation patterns of GP-V82 and GP-A82. We speculate that the acquisition of Le^x^ by GP-V82, particularly on glycans attached to Asn257 and Asn563, optimally positioned for engagement of a lectin CRD dimer, may enhance its interaction with DC-SIGN. The acquisition of the high-affinity Le^x^ DC-SIGN ligand and the increased accessibility of the glycans are likely to boost the binding of GP-V82 to DC-SIGN. Such multimeric interactions would stabilize viral attachment and promote more efficient infection of DC-SIGN-expressing cells, such as DCs.

This study has several limitations. First, no authentic EBOV was used in the experiments. Although lentiviral pseudoviruses are valuable tools that enable the safe study of EBOV outside BSL-4 conditions, they only partially recapitulate the wild-type virus, as they are replication-deficient and exhibit morphological differences. Some of these limitations are mitigated by the incorporation of FLPs, which provide a model that is morphologically closer to the authentic virus. In particular, the purified population enriched in filamentous particles, which are morphologically more similar to the authentic virus, exhibited increased infectivity when incorporating the GP-V82 variant compared with the GP-A82 variant. Furthermore, pseudoviruses allow further mutagenesis and detailed dissection of EBOV GP, enabling the study of differential interactions between GP-A82 and GP-V82 with DC-SIGN, analyses that would not be possible using authentic EBOV. The procedures described here also provide a useful framework for exploring the relationship between GP stability and glycan composition using GP mutants. This hypothesis is supported by reported observations for the GP-V82 variant, but it remains speculative and will require further experimental investigation to establish a mechanistic connection.

Another limitation is that evaluation of this phenomenon in physiologically relevant cell models is limited to primary MDDCs and M2-MDMs. Investigating GP-A82 and GP-V82 binding and infection in more complex systems, such as human organoids or humanized animal models expressing human DC-SIGN, would provide a more physiologically relevant evaluation of the mutation’s impact. Furthermore, it remains unclear whether the glycosylation conferring DC-SIGN-mediated enhancement to GP-V82 is maintained in more complex in vivo systems, and whether this phenotype is restricted to infection of specific host cells. The cell type producing the virus strongly dictates its N-glycosylation profile. Previous studies have shown that EBOV-GP produced in macrophages tends to acquire complex N-glycans, resulting in less efficient DC-SIGN-mediated entry compared with pseudoviruses bearing GP produced in other cell types or in the presence of the α-mannosidase inhibitor DMJ [62]. Therefore, the DC-SIGN-mediated enhancement we observe may vary during in vivo infection depending on the cellular origin of the circulating virions.

EBOV is primarily transmitted through skin or mucosal contact with infected body fluids. DCs and macrophages serve as the initial targets of infection, and then remain as central, sustained cellular targets throughout the entire disease course [63, 64]. Our findings reveal that the GP-V82 variant responsible for the 2013-2016 epidemic acquired distinctive glycosylation patterns that significantly increased its avidity for DC-SIGN, a lectin receptor highly expressed on mucosal DCs. This suggests that EBOV may have adapted to humans by optimizing interactions with a key cell surface receptor, thereby enhancing its ability to target and infect primary immune cells at mucosal entry sites. This adaptation likely conferred a selective advantage during human-to-human transmission, facilitating more efficient viral dissemination early in infection. Although the EBOV Makona variant spread extensively during the epidemic, the precise contribution of host cell receptors to transmission efficiency remains underexplored in current EBOV models. Our study provides compelling evidence that EBOV can exploit changes in envelope glycosylation to enhance receptor-mediated entry, underscoring the importance of glycan-lectin interactions in viral pathogenesis. These findings position DC-SIGN not only as an important host factor for EBOV entry but also as a potential driver of viral adaptation to humans. This mechanism may represent a broader evolutionary strategy employed by zoonotic viruses to optimize transmission in new hosts, highlighting glycosylation as a key determinant in cross-species transmission and epidemic potential.

## Online methods

### Vectors and recombinant DNA

The GP gene from an early isolate of EBOV Makona (GenBank accession no. KM233102.1) containing the naturally occurring V82 substitution was synthesized and cloned into pcDNA3.1 by GeneArt AG technology (Life Technologies). Site-directed mutagenesis was carried out by the Q5 Site-Directed Mutagenesis standard protocol (New England BioLabs). To prepare GPΔm-A82 and GPΔm-V82 without the MLD, GP residues 308 to 491 were removed, whereas glycosylation mutants were generated by single amino acid substitutions V82A, N563A, N563D, N238Y, N257D, N296D and N228D in the coding sequence of Makona GP-A82 or GP-V82. Primers were designed using NEBase Changer (New England Biolabs) software. Mutagenesis was verified by sequencing of the whole GP coding region. The pNGVL vector was used for expression of the VSV-G protein.

### Cell lines

Human embryonic kidney cells (293T/17; ATCC-CRL-11268) and human cervical adenocarcinoma cells (HeLa; ATCC-CCL-2) were cultured in Dulbecco’s modified Eagle medium (DMEM) supplemented (Gibco, Thermo Fisher) with 10% heat-inactivated fetal bovine serum (HI-FBS), 25 μg/mL gentamycin and 2 mM L-glutamine. Jurkat, Jurkat-DC-SIGN cells, Jurkat DC-SIGN-GFP [29], Raji and K562 were maintained in RPMI 1640 medium supplemented with 10% HI-FBS, 25 μg/mL gentamycin and 2 mM L-glutamine. Raji-DC-SIGN and K562-DC-SIGN [33] were maintained in RPMI 1640 medium supplemented with 10% HI-FBS, 25 μg/mL gentamycin, 2 mM L-glutamine and 200 μg/mL G418. All cells were incubated at 37°C with 5% CO_2_.

### Production of human macrophages and DCs

Blood samples were obtained by venipuncture into heparin tubes from healthy human donors under informed consent and IRB approval. Peripheral blood mononuclear cells (PBMCs) were isolated from buffy coats by Ficoll density gradient centrifugation (Histopaque; Sigma-Aldrich). To generate DCs, 2 × 10^6^ PBMC/mL were placed into 24-well plates and incubated for 1 h at 37°C with 5% CO_2_. The adhered monocytes were then washed twice with PBS and resuspended in RPMI supplemented with the cytokines GM-CSF (200 ng/mL) and IL-4 (10 ng/mL) (ImmunoTools). Differentiation to immature monocyte-derived DCs (MDDCs) was achieved by incubation at 37°C with 5% CO_2_ for 7 days and subsequent activation with cytokines on days 2 and 5. To generate M2 monocyte-derived macrophages (M2-MDMs), CD14^+^ monocytes were purified using anti-human CD14 antibody-labelled magnetic beads and iron-based LS columns (Miltenyi Biotec) and used immediately for further differentiation into macrophages [32]. CD14^+^ cells were cultured for 7 days in the presence of M-CSF (ImmunoTools), with cytokine addition every other day.

### Production of GP-pseudotyped lentiviral particles

Standard production of GP-pseudotyped lentiviral particles was performed in HEK-293T. Cells were plated in 10 cm tissue culture dishes (3 × 10^6^ cells/dish) and the next day transfected with 18 μg of pNL4-3.Luc.R^–^.E^–^ (NIH AIDS Reagent Program from Dr. Nathaniel Landau) [65] and 4 μg of the different EBOV GP or VSV-G expression vectors using either a standard calcium chloride transfection protocol or Lipofectamine 3000 transfection protocol (Invitrogen), according to manufacturer instructions. 16 h post infection, transfection medium is removed and 7 mL of fresh OptiMEM medium is added to the cells. Supernatants were recovered at 48 h post-transfection, cell debris was removed by centrifugation, and stored in aliquots at −80 °C. Infectious titres of the lentivirus-containing supernatants were titrated in HeLa cells as tissue culture infectious doses (TCID_50_) per mL by limiting dilution (1:5 serial dilutions in triplicates) or via luciferase activity measured as RLU in 96-well plates with 4 × 10^4^ HEK-293T cells per well, which was determined following a Luciferase Assay System in a GloMax®-Multi + Detection System (Promega). Briefly, pseudovirus stocks were titrated by infecting HEK-293T cells with serial dilutions of virus-containing supernatants and measuring luciferase activity as relative light units (RLU). For each stock, the relationship between the volume of the infectious supernatant and the resulting RLU signal was assessed across the serial dilution range. Subsequent infection experiments were performed using pseudovirus doses selected within the linear range of the assay, avoiding both background-level and saturated signals.

Production of GP-pseudotyped lentiviral particles in K562 was performed in 6-well plates (2 × 10^6^ cells/well). Transfection with 2 μg of pNL4-3.Luc.R^–^.E^–^ per well and 0.5 μg of the different EBOV GP expression vectors per well was performed using Lipofectamine 3000 transfection protocol (Invitrogen), according to manufacturer instructions. Supernatants were recovered at 48 h post-transfection, cell debris removed by centrifugation and stored in aliquots at −80 °C until use. Titration was performed measuring RLUs as described above.

### Infectivity assays with EBOV GP-pseudotyped particles

M2-MDM and MDDC (1 × 10^5^ cells/well) were infected with viral particles of Makona GP-A82 or GP-V82 pseudoviruses in a 48 well plate using the same amount of p24 antigen quantified by AlphaLISA (AL291C:500 assay; PerkinElmer). After 48h of incubation cells were washed twice with PBS and lysed for luciferase assay. For Jurkat-DC-SIGN, Jurkat DC-SIGN-GFP, Raji-DC-SIGN and K562-DC-SIGN and their controls (Jurkat, Raji and K562 cells), 1.5 × 10^5^ cells were infected with equal titres (50.000 RLU measured in 4 x10^4^ 293T-cells per well) of Makona GP-V82, GP-A82 and single N-linked glycosylation mutant pseudoviruses in a 96-well white plate (Corning). For comparison between GP-V82, GP-A82, GPΔm-A82 and GPΔm-V82, the virus particles were first concentrated by ultracentrifugation over a 20% sucrose cushion. Ultracentrifugation was performed at 28,000 x g for 2 h at 4°C in an Sorvall wX + Ultra Series Centrifuge (Thermo Scientific). After ultracentrifugation, they were resuspended in 1 mL of PBS, and 2 µg of total protein, as measured by microBCA (Thermo Fisher Scientific), was used for infection. After 48 h of incubation, infectivity was determined from the luciferase activity for wild type and mutant pseudoviruses, monitored by the luciferase assay described above. Overall, standardization of the infective units by p24 levels, RLUs or total protein led to comparable infectivity of GP-A82 or GP-V82 in HEK-293T cells.

For endoglycosidase treatments, ultracentrifuged pseudovirus were digested with Endo H (New England Biolabs) following manufacturers’ instructions for non-denaturing conditions. In brief, 5 µg of GP-A82 or GP-V82 pseudotyped lentivirus were digested with 2 µL of Endo H and 2 µL of GlycoBuffer 3 (10X) in a final volume of 20 µL and incubated overnight at 37°C. Then, infections were performed as described above.

For neutralization with anti-Le^x^ antibody (BD Pharmigen^TM^ Purified Mouse Anti-Human CD15, Clone MMA), pseudovirus were incubated at 37°C for 1 hr with anti-Le^x^ antibody at 10 µg/mL. Then, infections were performed as described above.

In all infection assays, infectivity was expressed as relative infection by normalizing the RLU signal of each virus sample to the mean signal obtained with GP-A82 within the same cell line, which was set as 100%. Comparisons between GP-A82 and GP-V82 were based on these normalized infectivity values obtained under matched experimental conditions.

### Virion-cell binding assay

To quantify the amounts of viral particles attached to the cell surface, 2 × 10^5^ Jurkat or Jurkat-DC-SIGN cells were chilled to 4 °°C before the addition of cooled virions (normalized by HIV p24Gag). The mixtures were spun at 2000 rpm for 1 h at 4 °°C as described [66], then cells were washed five times with RPMI at 4 °°C (to eliminate unbound viral particles), resuspended in 100 µL of PBS, and cell-associated p24 levels were quantified by AlphaLISA to determine binding.

### EBOV GP-VP40-Bla filovirus-like particles

To generate the EBOV GP-VP40-Bla FLPs, 80-90% confluent T-175 plates of HEK-293T cells were transfected using Lipofectamine 3000 with 40 μg of pcDNA3.1-BlaVP40 and 20 μg of the EBOV GP-A82, GP-V82 plasmids or none. Supernatants were harvested after 72 h of incubation and FLPs were concentrated by ultracentrifugation over a 20% sucrose cushion. Ultracentrifugation was performed in an Sorvall wX + Ultra Series Centrifuge with a AH629 rotor (ThermoFisher) at 28,000 rpm for 2 h at 4°C. After ultracentrifugation, the FLPs were resuspended in 1 mL of PBS. Protein concentration measured by microBCA (Thermo Fisher Scientific) following manufacturer instructions was used to estimate FLP concentration. Levels of GP and VP40 incorporated into 1 μg of FLPs bearing either GP-A82, GP-V82 or no GP (“Empty”) as measured by ELISA with optical density at 450 nm (OD450). Briefly, ELISA plates were coated with 1 μg per well of FLPs in 50 μL per well of PBS overnight at 4°C. Then, plates were washed three times with 200 μL PBS with 0.05% (v/v) of Tween-20 (PBS-T). Plates were blocked with 200 μL per well of 3% fat-free milk diluted in PBS-T for 1 h at room temperature (RT). GP and VP40 were detected with a 100 μL per well of rabbit polyclonal antibody GP- or VP40 -specific, respectively, from IBT bioservices, diluted in 3% milk in PBS-T at a final concentration of 0.1 μg/mL. Incubation was performed for 1.5 h at RT. Then, plates were washed three times with 200 μL PBS-T. 100 μL per well of a 1:3000 dilution goat anti-Rabbit IgG HRP secondary antibody (Thermo Fisher) for 1 h at RT. After three washes with 200 μL PBS-T, ELISA plates were incubated for 10 min with 50 μL 1-Step Ultra TMB-ELISA and then blocked with sulphuric acid (2M). Absorbance at 450 nm was measured.

Infection was performed as previously described, with some modifications [67]. In brief, cells were plated in a 96-well plate as described above (4 x10^4^ 293T cells per and 1.5 × 10^5^ K562 or K562-DC-SIGN cells per well), then they were infected with 10 μg per well of FLPs in a final volume of 200 μL of OptiMEM. Cells were centrifuged at 450 g for 60 min at 4°C to synchronize the infection (spinoculation). Then, cells were transferred to the incubator at 37°C and further incubated for 3 hr. To study the kinetics of FLPs cell entry, endocytic trafficking was stopped with 100 μM of hydroxychloroquine at different times after spinoculation: 0, 20, 40, 60, 90, 120 and 150 min. To detect Bla activity by flow cytometry, adhered cells were detached with trypsin and loaded with the CCF2-AM substrate (Invitrogen) for 45 min to 1 h at room temperature according to the manufacturer's instructions. Following substrate loading, cells were washed with PBS with 0.5% FBS and 1 mM CaCl_2_ and transferred to a round bottom 96-well plate. Cells were analyzed with a MacsQuant-10 Flow cytometer (Miltenyi Biotech), typically collecting 10,000-25,000 events. Samples were analyzed for their cleavage of CCF2 using FlowJo v10.0.0 software (Tree Star Inc.) and FCS Express v7.30.0018 (De Novo Software).

To enrich for filamentous FLPs, concentrated particle preparations were fractionated using a discontinuous sucrose step gradient, as has been described before [39, 40]. Briefly, FLPs were layered onto a gradient consisting of 4 mL of each 10%, 30%, and 60% sucrose layers. Following ultracentrifugation at 28,000 rpm as described above for 18 hr, 2 mL fractions (F1 to F6) were collected dropwise by piercing the bottom of the tube. The particles in the fractions were concentrated with a 100 K Amicon (Sigma Aldrich) and buffer was exchanged to PBS to an approximate final volume of 200 μL per fraction. Total protein concentration across fractions was quantified via micro-BCA, and the presence of EBOV GP1 and VP40-Bla was confirmed by SDS-PAGE. The enrichment of filamentous FLPs in the fractions was assessed by electron microscopy as described below. 2 μg/well of total protein from each fraction (F1-F4 were used, F5 and F6 were excluded due to insufficient protein concentration) were then used to infect K562-DC-SIGN cells to assess entry efficiency, as described above.

### Electron microscopy of the FLPs

Electron microscopy images of FLPs were acquired using the FEI Verios 460 SEM operated in scanning transmission mode (STEM) at 30 kV. Samples were deposited onto carbon film TEM grids (400 mesh copper, CF400-CU, Electron Microscopy Sciences) and stained with Uranyless (Delta Microscopies) to enhance contrast.

### Measurement of DC-SIGN expression

To evaluate DC-SIGN expression on the cell surface of MDDCs, MDM-M2 and transduced cells, they were stained with 2 μL of a PE-labelled anti-human CD209 (DC-SIGN) antibody (Clone 9E9A8, Biolegend) and 1 μL Viobility™ 488/520 Fixable Dye in a final volume of 100 μL of 0.5% FBS and 1 mM CaCl_2_ for 45 min at 4°C. A PE-labelled anti-HisTag antibody (Clone J095G46, Biolegend) was used as isotype control. Then cells were washed twice with 250 μL of 0.5% FBS and 1 mM CaCl_2_ and transferred into FACS tubes. Cells were analyzed with a MacsQuant-10 Flow cytometer (Miltenyi Biotech), typically collecting 10,000 events and analyzed using FlowJo 10.0.0 software.

### Protein production and purification

To produce the EBOV Makona GP ectodomains, the cDNA coding for residues S32 to T634 of the EBOV Makona variant [68], KM233069 sequence, was amplified by PCR and cloned into a pcDNA 3.1 vector (pcDNA/GP-V82), in frame with the IgK leader sequence and the HA epitope at the 5’ end, and with the T4 fibritin trimerization sequence, a FLAG peptide and a His-tag at the 3’ end. Constructs with the GP A82 residue (pcDNA/GP-A82) or those with the mucin domain (residues S312 to N462) replaced by the GSG sequence (GPΔm) were derived from pcDNA/GP-V82 by PCR following standard procedures. Soluble proteins were produced by transient transfection of suspension-adapted FreeStyle 293-F cells (ThermoFisher) cells and the amount of protein in the cell supernatants was monitored by sandwich ELISA with FLAG and biotin-labelled HA Abs. To prepare the (^13^C)-labelled GPΔm, 1.5 g of (^13^C)-glucose was added to 0.5 L of cell culture media before the cell transfection. The proteins were first purified with Nickel-agarose beads (ABT), and the eluted proteins with 250 mM imidazole in Tris-buffer saline (TBS, 20 mM Tris-HCl, 200 mM NaCl, pH 8.0) were concentrated and run through a Superose 6 (10/300) column in TBS or PBS. The elution profile of the proteins in the size exclusion chromatography was consistent with homotrimers. The extracellular domain (ECD) of DC-SIGN was produced and purified as described in Tabarani et al. [69].

### NMR data collection

After purification, the (^13^C)-labelled GPΔm-A82 and GPΔm-V82 glycoproteins were transferred to PBS buffer in D_2_O and concentrated to 0.27 mM and 0.21 mM, respectively. High-resolution ^1^H-^13^C-CT-HSQC spectra were acquired for both samples in a Bruker 700 MHz spectrometer equipped with a cryo-probe, with a constant t1(^13^C) time evolution of 19.4 ms and 240 scans. 2048 and 984 data points were acquired for F2 (^1^H) and F1 (^13^C) dimensions, respectively.

### Surface plasmon resonance interaction assays

Direct interaction studies were performed on the Biacore T200 at 25°C. DC-SIGN ECD surfaces were functionalized on a CM3 Sensorchip Series S (Cytiva) in HBS-P + buffer. Surface Fc1 was activated with a flow of 5 µL/min using 0.2 M EDC/0.05 M NHS mixture for 420 sec. Streptavidin was then injected at 100 µg/mL in 10 mM NaOAc pH 4 for 600 sec, followed by the injection of 1 M ethanolamine for 420 sec. The surface was washed for 60 sec with 10 mM HCl and with a mixture of 1 M NaCl and 50 mM NaOH at 100 µL/min to remove non-specific binding. A final streptavidin density of 1528 RU was reached. The Fc2 surface has been functionalized in the same way, reaching a streptavidin density of 707 RU. DC-SIGN ECD selectively biotinylated on its N-terminus as described by Achilli et al. [70] was captured on Fc2 streptavidin surface. The injection was performed at 0.1 µg/mL, reaching 143 RU of DC-SIGN surface density. Fc2 was then washed with 1 M NaCl and 50 mM NaOH for 12 seconds at 100 µL/min to remove non-specific binding. Once the functionalization was finished, the running buffer was replaced for 25 mM Tris pH 8.0, 150 mM NaCl, 4 mM CaCl_2_, 0.05% P20 surfactant.

Decreasing concentrations of EBOV GPΔm-A82 and GPΔm-V82 proteins were prepared in 25 mM Tris pH 8.0, 150 mM NaCl, 4 mM CaCl_2_, 0.05% P20 surfactant, ranging from 1.5 µM to 0.36 nM through a 1:2 serial dilution. 180 µL of each protein were injected in duplicate onto DC-SIGN ECD surfaces at a flow rate of 20 µL/min. Surface regeneration was performed with 50 mM EDTA injections at 100 µL/min for 10 seconds. Data were analyzed with the BiaEvaluation software through a steady state affinity model, and binding RU determined.

### Structure modelling and representations

The structure of an EBOV GP crystallized in the absence of ligand (PDB ID: 5JQ3) was used to generate the structural representations [71]. This high-resolution crystal structure (2.2 Å) of an EBOV GP-A82 variant provides a detailed view of the interaction network between N-gly563 and surrounding protein residues. It was selected instead of the more recent crystal structure of the EBOV Makona GP-V82 variant (PDB ID: 6VKM) [4], which was determined at lower resolution (3.5 Å). Structural superposition of the two structures showed that they are highly similar (RMSD  =   0.329 Å), with nearly identical conformations of the α1 helix containing the A82V substitution and adjacent residues.

A Man-5 glycan was modelled into the N-linked glycosylation sites defined in the crystal structure by superimposition of the chitobiose cores. To model the dimeric GP binding to DC-SIGN, two types of lectin dimers were generated first with the DC-SIGN molecule in complex with GlcNAc2Man3 (PDB ID 1K9I), based on the tetrameric DC-SIGNR crystal structure (PDB ID 1XAR). In one of the lectin dimers shown in Figure 4(E), the distance between the two carbohydrate-recognition sites was very similar to the distance between the glycans linked to Asn257 and Asn563 (∼40 Å). The β-mannoses bound to the two lectin monomers were superimposed simultaneously onto the β-mannoses of the Ngly257 and Ngly563, which resulted in the proposed dimeric interaction shown in Figure 4(E). Figures were prepared with PyMOL version 2.5.0 (pymol.org)

### Statistical analysis

Experimental data from infection and binding assays are presented as individual data points in the graphs. The number of independent experiments and biological replicates is specified in the figure legend, and error bars represent the standard deviation of the mean for linear scales and geometric standard deviation for logarithmic scales. Statistical tests performed in each experiment are described in the corresponding figure legend. Statistical analyses were conducted using GraphPad Prism software (version 10.0).

## Supplementary Material

Supplemental Material
