# Supplementary material for: Differential glycosylation in the envelope glycoprotein enhances DC-SIGN-mediated cell entry of the West African Ebolavirus Makona
Source: Emerg Microbes Infect. 2026 Sep 22;15(1):2732697. doi: 10.1080/22221751.2026.2732697 (PMC13600303; doi:10.1080/22221751.2026.2732697)
Supplement: Supplementary file 1 [file TEMI_A_2732697_SM3570.docx]

**SUPPLEMENTARY FIGURES AND LEGENDS**

**Figure S1. Sequence alignment of filovirus glycoproteins.** The extracellular mature region without the signal sequence has been included, with conserved residues highlighted in red. A82 in magenta and V82 in the Makona strain in yellow. Secondary structure elements based on the EBOV GP structure (PDB ID 5JQ3) are shown in orange (GP1) or green (GP2). The mucin-liked domain Mucin domain absent in the structure comprise residues 312 to 464, approximately. N-linked glycosylations sites in the EBOV GP are marked with a triangle below the sequences. Figure prepared with ESPript 3.0 ([http://espript.ibcp.fr](http://espript.ibcp.fr/)).

**Figure S2. Infectivity of pseudovirus bearing GP with or without the mucin-like domain (MLD) in different cell lines.** Infection levels of pseudovirus with GP-A82, GP-V82, GP∆m-A82 or GP∆m-V82 of different cell lines. Infection medium without pseudovirus was used as negative control. Relative luminescence units (RLUs) monitoring the infection of the indicated cell expressing or lacking cell surface DC-SIGN. Geometric mean ± geometric SD are plotted, with individual replicates represented by individual points, from three independent experiments. Comparison of infectivity between GP and GP∆m (significance values in red) or between GP-A82 and GP-V82 (significance values in black) in cells lacking or having DC-SIGN, respectively, was determined by a Mann Whitney U-test. Significance shown as: *p = 0.05 to 0.01, **p = 0.01 to 0.001, ***p = 0.001 to 0.0001, ****p < 0.0001.

**Figure S3. Infectivity of pseudovirus produced in K562 cells. A)** Infection levels of pseudovirus bearing GP-A82 or GP-V82 produced in K562 cells, measured as RLUs as in Figure S2. Geometric mean ± geometric SD of five replicates were plotted. **B)** Relative infection (%) of GP-V82 with respect to GP-A82. Mean ± SD of five replicates were plotted. Comparison between GP-A82 and GP-V82 infectivity was performed using a Mann Whitney U-test, and the significance is indicated as: *p = 0.05 to 0.01, **p = 0.01 to 0.001.

**Figure S4. Incorporation of GP-A82 and GP-V82 into the FLPs.** Levels of GP and VP40 incorporated into FLPs bearing either GP-A82, GP-V82 or no GP (“Empty”) as measured by ELISA by optical density at 450 nm (OD_450_). ELISA plates were coated with 1 μg per well of FLPs. GP and VP40 were detected with a rabbit polyclonal antibody (pAb, IBT bioservices) against GP or VP40.

**Figure S5. Enrichment of filamentous FLPs by sucrose gradient and DC-SIGN-dependent cell entry. A)** Schematic representation of the discontinuous sucrose step gradient (10%, 30%, and 60%) used for FLP fractionation. The resulting collected fractions are denoted as F1 to F6. **B)** Total protein concentration (µg/mL) quantified by microBCA across the collected fractions (F1–F6) for FLP preparations bearing either EBOV GP-A82 (blue) or GP-V82 (red). **C)** SDS-PAGE analysis of the collected fractions. Arrows indicate the bands corresponding to the EBOV GP1 (~75 kDa) and VP40-Bla (~70 kDa) proteins, which co-migrate and are predominantly enriched within the denser fractions (F2–F4), consistent with the expected sedimentation profile of filamentous particles. **D)** FLP fractions (F1-F4) infection of K562-DC-SIGN cells. The percentage of positive, infected cells was determined as described in Figure 2, with K562-DC-SIGN cells after 1 h of spinoculation with 2 μg of FLPs at 4ºC and after 3 h of incubation at 37ºC. Bars represent the mean + SD of three replicates, represented by individual dots. Statistical comparisons between GP-A82 and GP-V82 infectivity for each fraction was performed using a Two-way ANOVA, followed by a Šidák's multiple comparisons test. Significance is indicated as: *p ≤ 0.05, **p = 0.01 to 0.001.

**Figure S6. Conformation of the glycan N-linked to Asn563 (Ngly563) that shields the helix a1 bearing the A82V substitution.**  Ribbon and stick representations of the glycan and surrounding protein region in the crystal structure of the EBOV GP (PDB ID 5JQ3). The Asn563 in the GP2 HR1 helix and the N-linked glycan residues are shown as sticks with carbons in cyan, whereas the protein residues that contact the glycan are shown as sticks with carbons in orange (GP1) or green (GP2). Water molecules shown as red spheres, nitrogens in blue and oxygens in red, with hydrogen bonds represented as black dashed lines.

**Figure S7. Expression and incorporation of the EBOV GP glycosylation mutants into pseudovirus particles.** Western blot of GP without (WT) or with the indicated substitution in Asn residues at glycosylation sites (see Figure 4). Proteins were resolved by SDS-PAGE 12% and detected with a GP-specific antibody. Equal particle numbers (p24 based) were loaded in each track. GP was quantified with Image Lab 6.0.1 (BioRad); the relative GP (%) amounts in the particles with respect to the WT proteins are shown below the Western blot.

**Figure S8. Spectrum of the Neu5Ac units in the EBOV GP**. ^1^H-^13^C HSQC expansion of the region containing the H3 protons corresponding to the indicated carbohydrates in the overlaid spectra of GP-A82 spectrum (blue) and GP-V82 (red), shown as in Figure 5.

**Figure S9. Neutralization of lentivirus bearing GP-A82 or GP-V82 with an anti-CD15** (Le^x^) **antibody.** Percentage of neutralization of the pseudovirus incubated with 1:2 serial dilutions of the anti-CD15 antibody, starting at 10 µg/mL, was determined in HEK-293T (left) and Jurkat-DC-SIGN (right) cells. Mean ± SD of two replicates were represented.
